# Screening of Novel Pharmacogenetic Candidates for Mercaptopurine-Induced Toxicity in Patients With Acute Lymphoblastic Leukemia

**DOI:** 10.3389/fphar.2020.00267

**Published:** 2020-03-20

**Authors:** Minyuan Cao, Dandan Yin, Yun Qin, Fei Liao, Yali Su, Xuyang Xia, Ju Gao, Yiping Zhu, Wei Zhang, Yang Shu, Xiaoxi Lu

**Affiliations:** ^1^Department of Pediatric Hematology and Oncology, West China Second Hospital, Sichuan University, Chengdu, China; ^2^Department of Laboratory Medicine, Precision Medicine Center, State Key Laboratory of Biotherapy, West China Hospital, Sichuan University, Chengdu, China; ^3^Department of Radiology, West China Hospital, Sichuan University, Chengdu, China; ^4^Department of Clinical Pharmacology, Hunan Key Laboratory of Pharmacogenetics, Xiangya Hospital, Central South University, Changsha, China

**Keywords:** mercaptopurine, leukopenia, hepatoxicity, adverse drug reaction, pharmacogenetics, CYP2A7, NUDT15/TPMT widetype, COMT

## Abstract

A small proportion of patients with acute lymphoblastic leukemia (ALL) may experience severe leukopenia after treating with 6-mercaptopurine (6MP), which can be largely explained by germline variants in *TPMT* and *NUDT15*. However, a minority of patients who suffered such adverse drug reaction have *NUDT15*^wt/wt^*TPMT*^wt/wt^ genotype, indicating that other genetic factors may take part in. In this study, we genotyped 539 exon-located nonsilent pharmacogenetic variants in genes involved in phase I/II of drug metabolism in 173 pediatric patients with ALL and conducted association screening for 6MP-induced leukopenia. Besides *NUDT15* (rs116855232, *P* = 6.4 × 10^−11^) and *TPMT* (rs1142345, *P* = 0.003), a novel variant was identified in *CYP2A7* gene (i.e., rs73032311, *P* = 0.0007), which is independent of *NUDT15/TPMT* variant. In addition, a variant (i.e., rs4680) in COMT is significantly associated with 6MP-induced hepatotoxicity (*P* = 0.007). In conclusion, variants in *CYP2A7* and *COMT* may be considered as novel potential pharmacogenetic markers for 6MP-induced toxicities, but additional independent validations with large sample size and investigations on related mechanisms are further needed.

## Introduction

Thiopurine [e.g., 6-mercaptopurine (6MP)] is crucial for curative chemotherapy for pediatric patients with acute lymphoblastic leukemia (ALL), which is the most common childhood cancers around the world (Pui and Evans, 2006). However, 6MP may induce severe toxicities in a small proportion of patients with standard dosage because of its narrow therapeutic index, including hematotoxicity (e.g., leukopenia) and hepatotoxicity (Pui and Evans, 2006; Moriyama et al., 2015). Single-nucleotide polymorphism (SNP) in *TPMT* (i.e., rs1142345) was first identified as a pharmacogenetic marker through pharmacology-guided approach, explaining the majority of 6MP-induced leukopenia cases in Caucasians and Africans (Relling et al., 1999; Moriyama et al., 2015). Thereafter, genetics-guided 6MP dosage adjustment is applied clinically and largely reduced the incidence of such adverse drug reaction (ADR) (Relling et al., 2013; Relling et al., 2019). However, compared with Caucasians, allele frequency of rs1142345 is lower in East Asians (approximately 1%), which cannot explain the higher incidence of 6MP-induced leukopenia in such population. With the development of genomic technology, novel and an ethnic-specific pharmacogenetic marker in *NUDT15* (i.e., rs116855232) has been identified through genome-wide association studies (Yang et al., 2015; Moriyama et al., 2016) and validated by multiple independent studies. Allele frequency of rs116855232 is approximately 10% in East Asians, accounting for a large proportion of 6MP-induced leukopenia, as well as 6MP tolerance with high sensitivity and specificity in such population (Yin et al., 2017; Zhu et al., 2018; Relling et al., 2019; Schaeffeler et al., 2019). Ethnic specificity is observed for rs116855232, because allele of this variant is close to 0% in Caucasians and Africans, which is much lower than that in East Asians. After screening the whole coding region of *NUDT15* in patients, less frequent and rare variants in *NUDT15* were also identified to be related to 6MP-induced leukopenia, also exhibiting ethnic-specific manner (Moriyama et al., 2017; Zhu et al., 2018). Mechanically, TPMT can anabolize 6MP into the inactive methyl-mercaptopurine (Mcleod et al., 2000), whereas NUDT15 acts as a nucleotide triphosphate diphosphatase, catalyzing the hydrolysis of nucleoside triphosphates, including dGTP and its analogs (e.g., 6MP) (Moriyama et al., 2016). Variants in both genes induce loss of function of their enzymatic activity and the subsequent accumulation of toxic metabolites (i.e., thioguanine nucleotides), resulting in leukopenia (Moriyama et al., 2016). Combination of common and rare functional variants of these two genes can increase the sensitivity for predicting ADR events without largely impacting the specificity (Zhu et al., 2018). However, a minority of ALL patients with hematotoxicity carry wild-type genotypes of both *NUDT15* and *TPMT*, suggesting that other genetic factors may be involved in these patients.

On the other hand, several candidate hypothesis studies illustrated the modest impact of variant in *ITPA* on 6MP-induced hepatotoxicity (Adam De Beaumais et al., 2011), which exhibits inconsistency in independent patient cohorts. With genome-wide approach, rs738409 at *PNPLA3* was identified as a genetic marker to indicate 6MP-induced alanine aminotransferase increase in Caucasians (Liu et al., 2017), with independent validation only in a limited study with Spanish population (Gutierrez-Camino et al., 2017).

Multiple phases I and II enzymes are required for metabolism of most of small molecular drugs. According to the classic pharmacokinetics study, 6MP is an inactive prodrug, intracellularly activated by *HPRT1*, *IMPDH1*, and so on (Zimm et al., 1986; Kirschner-Schwabe et al., 2006), while inactivated by TPMT (a phase II enzyme). On the other hand, NUDT15, another inactivated enzyme for 6MP, was recently introduced as a phase I enzyme after identification of its involvement in 6MP-induced leukopenia. Indeed, lots of genetic variants in phase I/II genes have been reported to impact on activity their encoded enzymes and subsequent drug efficacy/safety and translated into genetics-guided precise medication clinically (Relling and Evans, 2015). Whether 6MP-induced leukopenia can be explained by these variants (besides those in *NUDT15* and *TPMT*) is largely unknown, particularly in patients with *NUDT15*^wt/wt^*TPMT*^wt/wt^ genotype.

In this study, we aim to identify independent pharmacogenetic markers of 6MP-induced toxicities through screening the reported variants in phase I/II genes with customer-designed microarray, so as to increase the predictive sensitivity of 6MP-related ADR.

## Methods

### Patients

This study consisted of 173 pediatric ALL patients prospectively enrolled onto the CCCG-ALL-2015 protocol-based trial (http://www.chictr.org.cn, identifier ChiCTR-IPR-14005706) from West China Second Hospital between 2015 and 2016. The same patient cohort has been described in our previous report, except that 15 patients were excluded because of the unavailability or quality control failure of their samples. 6-Mercaptopurine was used in the last 2 weeks of remission induction stage (standard dosage of 60 mg/m^2^), consolidation stage (25 mg/m^2^), and maintenance stage (50 mg/m^2^). Forty-seven and 36 patients experienced 6MP-induced leukopenia (decreased white blood cell/neutrophil counts) and hepatotoxicity (five folds of increased aspartate transaminase and/or alanine transaminase), respectively. This study was approved by the ethics committee of West China Second Hospital, Sichuan University, and informed consent was obtained from patients or their guardians, as appropriate.

### Genotyping and Quality Control

Genotyping of ALL cases was performed by using customer-designed microarray, containing probes against 539 nonsilent exon-located SNPs in 156 phase I/II genes. Genotype calls of these SNPs were determined by the standard algorithms. Samples with more than 5% of failed genotyped SNPs, as well as SNPs with more than 5% of failed genotyped samples, were excluded from the analyses. Quality control was conducted based on genotyping quality, call rate, minor allele frequency, and Hardy–Weinberg equilibrium of all SNPs. Finally, 253 SNPs were left for association analyses (**Supplementary Table 1**).

### Statistical Analyses

The association of genotypes at each SNP with 6MP-induced leukopenia or hepatotoxicity was tested by comparing the genotype frequency between patients who experienced ADRs and those who did not by using logistic regression model. Independent association was also conducted by using rs116855232 (*NUDT15*) and rs1142345 (*TPMT*) as covariants for all patients, as well as in *NUDT15*^wt/wt^*TPMT*^wt/wt^ patients (n = 133) with logistic regression.

## Results

### Screening of Pharmacogenetic Markers for 6MP-Induced Leukopenia

Consistent with our previous report, no significant association of clinical characteristic with 6MP-induced leukopenia/hepatotoxicity was found (**Table 1**). Next, we conducted screening for pharmacogenetics markers from all exon-located SNPs in reported genes involved in phases I and II drug metabolism. In total, 3 SNPs exhibit significant association with 6MP-induced leukopenia (*P* < 0.01, **Table 1**), including two well-reported variants at *NUDT15* (rs116855232, *P* = 6.37 × 10^−11^) and *TPMT* (rs1142345, *P* = 0.003), respectively. Interestingly, a novel variant (rs73032311) at *CYP2A7* was also identified (*P* = 0.0007), with risk allele frequency (RAF) of 26.1% versus 10.7% in patients with/without such ADR, respectively. Moreover, rs73032311 exhibits significance after adjusting for rs116855232 and rs1142345 (*P*_adj_ = 0.027), indicating its independence of predicting 6MP-induced leukopenia. Next, we estimated the impact of rs73032311 in patients who carry no risk allele of *NUDT15* and *TPMT* variants (n = 133) and established the significance in such subgroup of patients with *P* = 0.04. Risk allele frequency of rs73032311 in *NUDT15*^wt/wt^*TPMT*^wt/wt^ patient with 6MP-induced leukopenia is 27.3% compared to 10.3% in patients without such ADR (**Table 1**). Additionally, we noticed the *P* value of rs73032311 increased after adjusting for *NUDT15* and *TPMT* variants (*P* = 0.0007 vs. 0.02*)*, suggesting that rs73032311 may interact with these two variants. We therefore check the RAF of rs73032311 in patients with heterozygous genotype at either rs116855232 or rs1142345. Interestingly, patients with heterozygous genotype of *NUDT15* but not the *TPMT* variant may have a higher risk to experience 6MP-induced leukopenia when they also carry risk allele of rs73032311. In our study, 100% of patients with *NUDT15*^wt/mut^*CYP2A7*^mut/mut^ (n = 1) and 80% with *NUDT15*^wt/mut^*CYP2A7*^wt/mut^ (n = 15) genotypes experience 6MP-induced leukopenia, compared to 50% *NUDT15*^wt/mut^*CYP2A7*^wt/wt^ (n = 14). Finally, the combination of *NUDT15*, *TPMT*, and *CYP2A7* variants improves predictive sensitivity of 6MP-induced leukopenia from 70.7% to 85.4%, but decreases the specificity (91.7% vs. 70.7%).

**Table 1:** Association of clinical and genetic characteristics with 6MP-induced leukopenia.

Features	Leukopenia		*P*
	With (n = 47)	Without (n = 126)	
	Median ± SD		
Initial WBC (10^9^/L)	12.7 ± 96.36	10.2 ± 91.82	0.96
Age at diagnosis	4.8 ± 3.02	4.55 ± 3.18	0.99
No. of males (%)	**Number of patients (%)**		
	26 (55.3%)	62 (49.2%)	0.48
Genetic factor*	**Number of risk allele carriers** **(risk allele frequency)**		
*NUDT15*			
rs116855232(C/T)	32 (40.4%)	4 (1.6%)	6.37 × 10^−11^*
*TPMT*			
rs1142345(A/G)	7 (8.5%)	1 (0.4%)	0.0034*
*CYP2A7*			
rs73032311(T/C)	23 (26.1%)	25 (10.7%)	0.0007*

*P <0.05

### Screening of Pharmacogenetic Marker for 6MP-Induced Hepatotoxicity

First, we estimated the association of the reported variants with 6MP-induced hepatotoxicity in our patients, including rs1127354 in *ITPA* and rs738409 in *PNPLA3*. However, no significance was observed for either of these two variants, with *P* = 0.89 and 0.55, respectively. Therefore, screening was performed for 6MP-induced hepatotoxicity through the similar strategy described above. Only 1 of 253 variants integrated in our microarray has *P* < 0.01, located in *COMT* gene (i.e., rs4680, *P* = 0.006) (**Table 2**). Allele frequency of this SNP is 27.54%, with 40.3% of patients experiencing hepatotoxicity compared to 24.2% in those who do not. Taking patients with either heterozygous or homozygous of the risk allele into account, sensitivity and specificity are 52.9% and 66.7%, respectively.

**Table 2:** Association of clinical and genetic characteristics with 6MP-induced hepatotoxicity.

Features	Hepatotoxicity		*P*
	With (n = 36)	Without (n = 136)	
Initial WBC (10^9^/L)	12.7 ± 152.6	10 ± 81.17	0.23
Age at diagnosis	4.8 ± 3.34	4.70 ± 3.02	0.52
No. of males (%)	**Number of patients (%)**		
	21 (58.33%)	66 (48.53%)	0.3
Genetic factor*	**Number of risk allele carriers** **(risk allele frequency)**		
*COMT*			
rs4680(G/A)	24 (40.3%)	58 (24.2%)	0.007*

*P <0.05

### Prediction Accuracies of Variants for 6MP-Induced ADR

**Figure 1:**
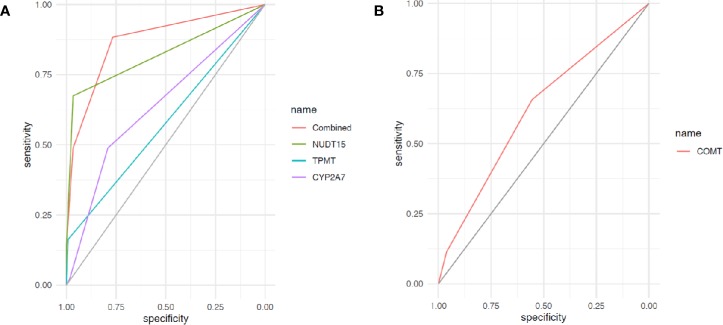
Receiver operating characteristic (ROC) curves of gene variants for 6-mercaptopurine (6MP)–induced ADRs. **(A)** Receiver operating characteristic curve of variants in *NUDT15*, *TPMT*, *CYP2A7*, and combinations for 6MP-induced leukopenia; **(B)** ROC curve of variants in *NUDT15*, *TPMT*, *CYP2A7*, and combinations for 6MP-induced hepatotoxicity.

Receiver operating characteristic curves were used to demonstrate the prediction accuracies for 6MP-induced leukopenia and hepatotoxicity in single variant as well as combination. Not surprisingly, rs116855232 in *NUDT15* exhibited the best prediction efficacy in a single gene prediction model. Area under the curve (AUC) for 6MP-induced leukopenia with this variant reached 0.82 [95% confidence interval (CI), 0.75–0.90], compared with 0.58 (95% CI, 0.52–0.63) for rs1142345 in *TPMT* and 0.63 (95% CI, 0.55–0.72) for rs73032311 in *CYP2A7*. After combining these three SNPs, the prediction accuracy increased with AUC of 0.87 (95% CI, 0.81–0.93) (**Figure 1A**). In another hand, AUC for 6MP-induced hepatotoxicity with rs4680 in *COMT* reached 0.62 (95% CI, 0.52–0.71) (**Figure 1B**).

## Discussion

With the rapid development of genomic techniques, strategies to screen pharmacogenetic markers have changed from pharmacology-based to genomics-based approaches, which is also considered as from candidate hypothesis to zero hypothesis. For instance, multiple novel pharmacogenetic biomarkers have been found by conducting genome-wide association approaches (Mccormack et al., 2011; Zhou et al., 2011; Xu et al., 2015). Recently, common variant in *NUDT15* (i.e., rs116855232) was identified to largely explain thiopurine-induced leukopenia in ALL and immune diseases (e.g., inflammatory bowel disease) in East Asians (Yang et al., 2014; Yang et al., 2015; Moriyama et al., 2016). Subsequent pharmacological analyses revealed the mechanism of how NUDT15 works in thiopurine metabolism (Moriyama et al., 2016). Additionally, rare variants that affect enzyme the activity of NUDT15 will also result in the 6MP-induced leukopenia (Moriyama et al., 2016; Moriyama et al., 2017; Zhu et al., 2018). Importantly, 6MP-induced leukopenia can also be validated in the mouse model (Nishii et al., 2018), indicating that it is practically to conduct this zero-hypotheses approach to identify pharmacogenetic markers. In this study, we focused on patients with *NUDT15*^wt/wt^*TPMT*^wt/wt^ genotype, in order to identify the novel variants in drug metabolism–related genes that can explain the rest of patients’ experience of 6MP-induced leukopenia. Finally, we found that rs73032311 is not only independently associated with such ADR, but also interacted with the variant in *NUDT15* but not *TPMT*. Statistically, variant in *CYP2A7* has a smaller *P* value as well as odds ratio than the variant in *TPMT*, probably because of its higher RAF. In addition, rs73032311 is a common SNP with varied RAF among different ethnicities, indicating that it may be served as a general pharmacogenetic marker. Importantly, variant in *NUDT15* exhibits the strongest prediction accuracy, and rs73032311 in *CYP2A7* has a higher accuracy than that of rs1142345 in *TPMT*, indicating the importance of the novel variant identified in this study for Chinese population. However, the limitation of this study is obvious due to the lack of validation cohort; independent replication studies are urgently needed, especially in patient cohorts with a large sample size. On the other hand, the clinical strategy should be carefully designed given that the significance of the CYP2A7 variant was validated, because the specificity was greatly decreased after introducing the novel variant. It will be more practical than genotyping rs73032311 in the first place, following up the risk allele carriers without reducing the dosage immediately. Functionally, amino acid alteration induced by rs73032311 of *CYP2A7* gene is predicted to be damaged according to CADD analysis, with the estimated PHRED score of 12.48 (rank as top 10%), suggesting its potential as a causal variant. Functionally, *CYP2A7* is considered to encode a member of the cytochrome P450 enzymes, which are well-reported to be involved in phase I of drug metabolism. Compared to the well-described P450 enzymes (e.g., CYP2D6), substrate to CYP2A7 has not yet been clearly determined. On the other hand, no p450 enzyme has been reported to be involved in thiopurine metabolism according to the current pharmacologic records. Considering *CYP2A7* is highly expressed in the liver, which is the main organ in which thiopurine is metabolized, the details on the mechanism of how the variant in *CYP2A7* impacts on 6MP-induced leukopenia are needed to be further elucidated.

On the other hand, no widely accepted pharmacogenetic biomarker has been recorded for 6MP-induced hepatotoxicity. Variants in *ITPA* and *PNPLA3* were reported to be associated with such ADR through candidate- or zero-hypothesis approaches, respectively (Adam De Beaumais et al., 2011; Liu et al., 2017). However, more replication studies are needed in additional patient cohorts with larger samples because of the inconsistency or limited validation, especially in other ethnicities (e.g., East Asians) (Steponaitiene et al., 2016; Gutierrez-Camino et al., 2017; Khera et al., 2019). In our study, neither rs1127354 in *ITPA* nor rs738409 in *PNPLA3* can be validated possibly due to the difference in patient cohort and ethnicities. Sample size may not be the major cause because no trend was observed with odds ratio of 1.32 and 1.18, respectively, which is much lower than that of rs4680 in *COMT*. Inconsistent association of these variants in our patients may be induced by their ethnic specificity; that is, the reported variants themselves may not be the functional cause for 6MP-induced hepatotoxicity, but only tag the causal variants through linkage disequilibrium, which is different among ethnicities. We thus performed screening variants in genes involved in phase I or phase II drug metabolism to find the potential pharmacogenetic markers for 6MP-induced hepatotoxicity in Chinese population. This is the first systematic screening in such ethnicity to the best of our knowledge. However, the accuracy for the novel variant is modest to predict 6MP-induced hepatotoxicity and thus is not efficient enough as a strong pharmacogenetic marker. Therefore, screening with a large sample size is needed, not only validating the significance of COMT variant, but also finding additional variants to further increase the predictive accuracy for 6MP-induced hepatotoxicity. Functionally, rs4680 in *COMT* has been widely reported to be associated with a variety of different drug reactions, labeling as level 2 pharmacogenetic biomarker in PharmGKB data resource (https://www.pharmgkb.org/) for efficacy of multiple drugs, including nicotine and morphine, without well-described mechanism (Sun et al., 2012; Ahlers et al., 2013). Functionally, COMT is universally expressed in multiple organs, including the liver; the details of how this gene is involved in 6MP-induced hepatotoxicity are also needed to be investigated after validation of the association of rs4680 with such ADR in independent patient cohorts.

## Data Availability Statement

The datasets generated for this study can be found in Array Express in the Genome Variation Map (GVM) database (http://bigd.big.ac.cn/gvm) with the accession number of GVM000060, and EBI database (https://www.ebi.ac.uk/arrayexpress/) with the accession number of E-MTAB-8613.

## Ethics Statement

This study was approved by the Ethics Committee of West China Second Hospital, Sichuan University, and informed consent was obtained from patients or their guardians, as appropriate.

## Author Contributions

XL and YS conceived the research project, wrote the manuscript, and gave final approval. MC, DY, YQ, FL, and XX analyzed and interpreted the data. MC, YLS, YZ, JG, and XL evaluated the patients and collected the clinical samples and data. WZ critically reviewed the manuscript.

## Funding

This work was supported by the National Natural Science Foundation of China (No. 81673452, No. 81903735, No. 81973408, and No. 81902872) and the National Key Research and Development Program of China (No. 2016YFC0905000 [2016YFC0905001]).

## Conflict of Interest

The authors declare that the research was conducted in the absence of any commercial or financial relationships that could be construed as a potential conflict of interest.

## Supplementary Material

Supplementary Table 1 Information of the raw genotype and p values for each SNP.
